# Two 3-amino-1*H*-pyrazol-2-ium salts containing organic anions, and an ortho­rhom­bic polymorph of 3-amino-1*H*-pyrazol-2-ium nitrate

**DOI:** 10.1107/S2056989020015959

**Published:** 2021-01-01

**Authors:** Sreeramapura D. Archana, Channappa N. Kavitha, Hemmige S. Yathirajan, Sabine Foro, Christopher Glidewell

**Affiliations:** a Department of Studies in Chemistry, University of Mysore, Manasagangotri, Mysuru-570 006, India; b Department of Chemistry, Maharani’s Science College for Women, Mysuru-570 001, India; c Institute of Materials Science, Darmstadt University of Technology, Alarich-Weiss-Strasse 2, D-64287 Darmstadt, Germany; d School of Chemistry, University of St Andrews, St Andrews, Fife KY16 9ST, UK

**Keywords:** pyrazoles, organic salts, crystal structures, polymorphism, hydrogen bonding, supra­molecular assembly

## Abstract

Co-crystallization from methanol of 3-amino-1*H*-pyrazole with 3,5-di­nitro­benzoic acid produces 3-amino-1*H*-pyrazol-2-ium 3,5-di­nitro­benzoate monohydrate, C_3_H_6_N_3_^+^·C_7_H_3_N_2_O_6_^−^·H_2_O, (I), while similar co-crystallization of this pyrazole with an equimolar qu­antity of fumaric acid produces bis­(3-amino-1*H*-pyrazol-2-ium) fumarate–fumaric acid (1/1), 2C_3_H_6_N_3_^+^·C_4_H_2_O_4_^2−^·C_4_H_4_O_4_, (II). The reaction of 3-amino-1*H*-pyrazole with a dilute solution of nitric acid in methanol yields a second, ortho­rhom­bic polymorph of 3-amino-1*H*-pyrazol-2-ium nitrate, C_3_H_6_N_3_^+^·NO_3_^−^, (III). In each of (I)–(III), the bond distances in the cation provide evidence for extensive delocalization of the positive charge. In each of (I) and (II), an extensive series of O—H⋯O and N—H⋯O hydrogen bonds links the components into complex sheets, while in the structure of (III), the ions are linked by multiple N—H⋯O hydrogen bonds into a three-dimensional arrangement. Comparisons are made with the structures of some related compounds.

## Abstract 2

The hydrogen-bonded assembly of 3-amino-1*H*-pyrazol-2-ium 3,5-di­nitro­benzoate monohydrate and bis­(3-amino-1*H*-pyrazol-2-ium) fumarate fumaric acid is two-dimensional, but that in the ortho­rhom­bic form of the simple salt 3-amino-1*H*-pyrazol-2-ium nitrate is three-dimensional.

## Chemical context   

**Figure 1:**
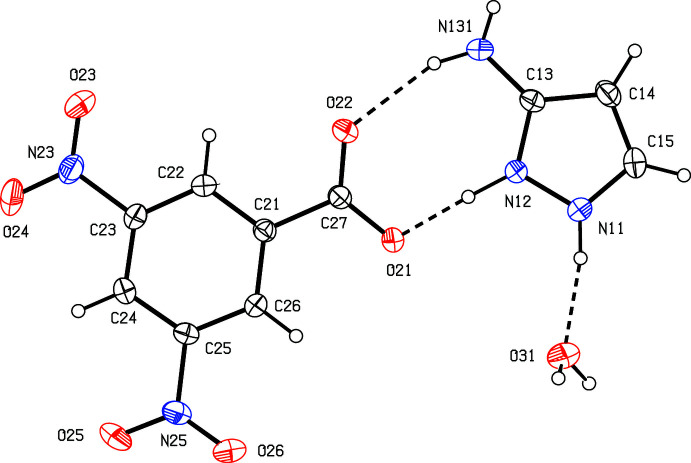
The independent components in compound (I) showing the atom-labelling scheme and the hydrogen bonds within the selected asymmetric unit. Displacement ellipsoids are drawn at the 30% probability level.

**Figure 2:**
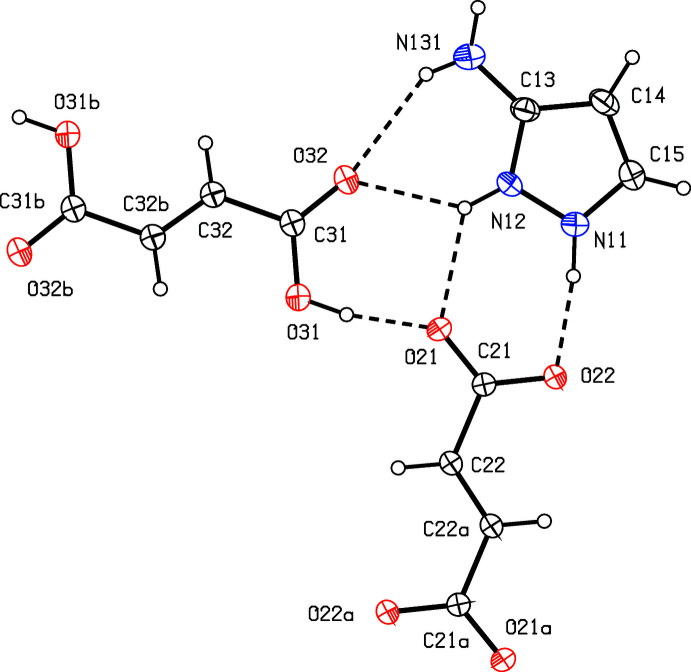
The independent components in compound (II) showing the atom-labelling scheme and the hydrogen bonds within the selected asymmetric unit. Displacement ellipsoids are drawn at the 30% probability level, and the atoms marked with the suffix ‘a’ or ‘b’ are at the symmetry positions (1 − *x*, 1 − *y*, 1 − *z*) and (1 − *x*, −*y*, 1 − *z*), respectively.

Pyrazoles exhibit a very wide range of pharmacological and other biological activities, which have recently been extensively reviewed (Ansari *et al.*, 2017 ▸; Karrouchi *et al.*, 2018 ▸). Derivatives derived from 3-amino-1*H*-pyrazole have been reported as tyrosine kinase inhibitors, of potential use in cancer treatment (Feng *et al.*, 2008 ▸) and as inhibitors of the intra­cellular phospho­rylation of the heat-shock protein hsp27 (Velcicky *et al.*, 2010 ▸). As part of a general study of novel pyrazole derivatives (Asma *et al.*, 2018 ▸; Kiran Kumar *et al.*, 2020 ▸; Shaibah *et al.*, 2020*a* ▸,*b* ▸; Shreekanth *et al.*, 2020 ▸), we have now synthesized two organic salts derived from 3-amino-1*H*-pyrazole, namely 3-amino-1-pyrazol-2-ium 3,5-di­nitro­benz­oate monohydrate (I) (Fig. 1 ▸ and Scheme) and bis­(3-amino-1-pyrazol-2-ium) fumarate fumaric acid (II) (Fig. 2 ▸), whose mol­ecular and supra­molecular structures are reported here. Compounds (I) and (II) were readily prepared by co-crystallization of 3-amino-1*H*-pyrazole with an equimolar qu­antity of the appropriate organic acid. We have also isolated a second polymorph of 3-amino-1-pyrazol-2-ium nitrate (III). When crystallized from methanol, this compound forms an ortho­rhom­bic polymorph in space group *Pna*2_1_; a monoclinic polymorph in space group *P*2_1_/*c*, isolated from aqueous solution has recently been reported (Yamuna *et al.*, 2020 ▸). Here we discuss the mol­ecular and supra­molecular structures of both polymorphs of the nitrate salt.
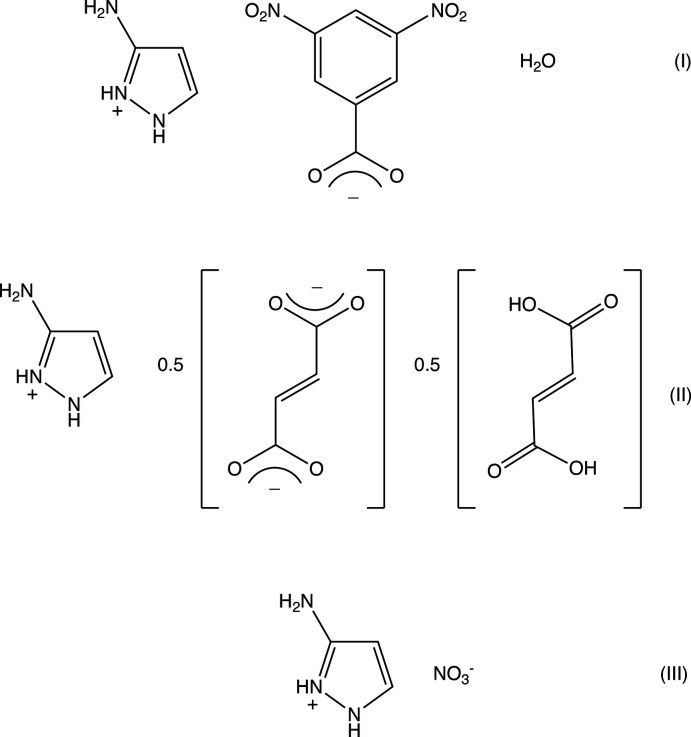


## Structural commentary   

The salt 3-amino-1*H*-pyrazol-2-ium 3,5-di­nitro­benzoate crystallizes from methanol as a monohydrate, although methanol is absent from the crystal structure. The constitution of the salt (I) derived from fumaric acid is more complex: the structure contains a single cation, occupying a general position, along with a fumarate dianion and a neutral fumaric acid mol­ecule, each lying across a centre of inversion, selected as those at (0.5, 0.5, 0.5) and (0.5, 0, 0.5), respectively, for the anionic and neutral components. The correct location of the H atom bonded to atom O31 (Fig. 2 ▸) was confirmed not only by refinement of the atomic coordinates for this H atom and by the final difference map, but also by the C—O distances in the two fumaric acid units, thus 1.2472 (17) and 1.2525 (15) Å in the anion, and 1.2136 (17) and 1.3065 (18) Å in the neutral fumaric acid mol­ecule. Although the co-existence of equal numbers of fumarate anions and neutral fumaric acid mol­ecules, as opposed to hydrogenfumarate anions, seems at first sight unexpected or even counter-intuitive, in fact a number of structures have been reported in which this combination is present, as noted below in Section 4.

Isolation of the nitrate salt from a methanol solution produces an ortho­rhom­bic form with space group *Pna*2_1_; it has recently been reported [Yamuna *et al.*, 2020 ▸; CSD (Groom *et al.*, 2016 ▸) refcode NUKKOW], that crystallization of the nitrate salt from an aqueous solution provides a monoclinic polymorph with space group *P*2_1_/*c*, which it is convenient to denote here as (III*a*). There is no obvious simple relationship between either the direct or the reduced cell dimensions for these two polymorphs.

For each of (I)–(III) it is possible to selected a compact asymmetric unit in which the components are linked by N—H⋯O hydrogen bonds (Figs. 1 ▸–3 ▸ ▸). Within the asymmetric unit of (II), there is a fairly short but markedly asymmetric O—H⋯O hydrogen bond (Table 2 ▸) linking the anionic and neutral fumaric fragments.

The bond distances within the cations exhibit some inter­esting features. In neutral 1*H*-pyrazole, the bonds corresponding to N12—C13 and C14—C15 in compounds (I)–(III) (*cf*. Figs. 1 ▸–3 ▸ ▸) are formally double bonds, while the other ring bonds are all formally single bonds. However, as shown in Table 1 ▸, which also includes data for the monoclinic polymorph (III*a*) (Yamuna *et al.*, 2020 ▸) for comparison, in none of the cations discussed here does the range of the C—N distances exceed 0.03 Å, while the difference between the two C—C distances never exceeds 0.04 Å. These observations indicate that the positive charge is delocalized over all three of the N atoms, such that all three canonical forms (*A*)–(*C*) (Fig. 4 ▸) are significant contributors to the overall electronic structure of the cation.

## Supra­molecular features   

The supra­molecular assembly in compounds (I)–(III) is dominated by N—H⋯O Hydrogen bonds together with O—H⋯O hydrogen bonds in (I) and (II) (Table 2 ▸). For the two-centre inter­actions, those having *D*—H⋯*A* angles significantly less than 140° have been discounted, as the associated inter­action energies are likely to be negligible (Wood *et al.*, 2009 ▸). Such contacts are better regarded as adventitious contacts that arise within the supra­molecular arrangements dominated by the significant hydrogen bonds.

The two ionic components in compound (I) are linked by two N—H⋯O hydrogen bonds, forming an 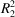
(8) ring (Etter, 1990 ▸; Etter *et al.*, 1990 ▸; Bernstein *et al.*, 1995 ▸), and a third N—H⋯O links the water component to the ion pair, forming a three-component aggregate (Fig. 1 ▸). The hydrogen-bonded supra­molecular assembly in compound (I) is two-dimensional. The O—H⋯O hydrogen bond involving atom H31 (Table 2 ▸) links the aggregates which are related by translation along [010] to form a 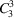
(9)
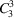
(9)[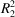
(8)] chain of rings. In addition, the N—H⋯O hydrogen bond involving atom H132 links the ion pairs that are related by translation along [001] into a 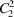
(10)
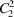
(12)[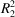
(8)] chain of rings. The combination of these two chain motifs generates a sheet lying parallel to (100) and containing 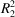
(8) and 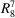
(32) rings (Fig. 5 ▸). Finally, the second O—H⋯O hydrogen bond involving atom H32 links pairs of such sheets, which are related by inversion, to form a complex bilayer.

The supra­molecular assembly in compound (II) is relatively straightforward. The single O—H⋯O hydrogen bonds links the fumarate ions and the fumaric acid mol­ecules into a chain running parallel to the [010] direction, in which the anions and neutral mol­ecules alternate (Fig. 6 ▸). Two chains of this type, which are related to one another by the *c*-glide planes, pass through each unit cell and they are linked by the cations, *via* a combination of N—H⋯O hydrogen bonds, to form a sheet lying parallel to (102), within which rings of 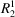
(6), 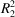
(6), 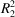
(7) and 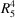
(22) types are present (Fig. 7 ▸).

**Figure 3:**
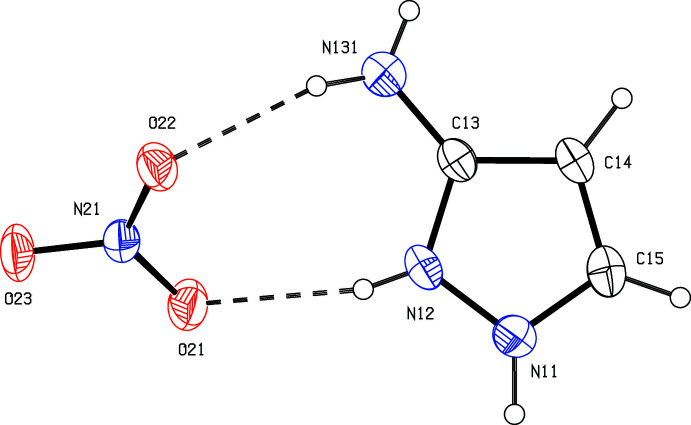
The independent components in compound (III) showing the atom-labelling scheme and the hydrogen bonds within the selected asymmetric unit. Displacement ellipsoids are drawn at the 30% probability level.

**Figure 4:**
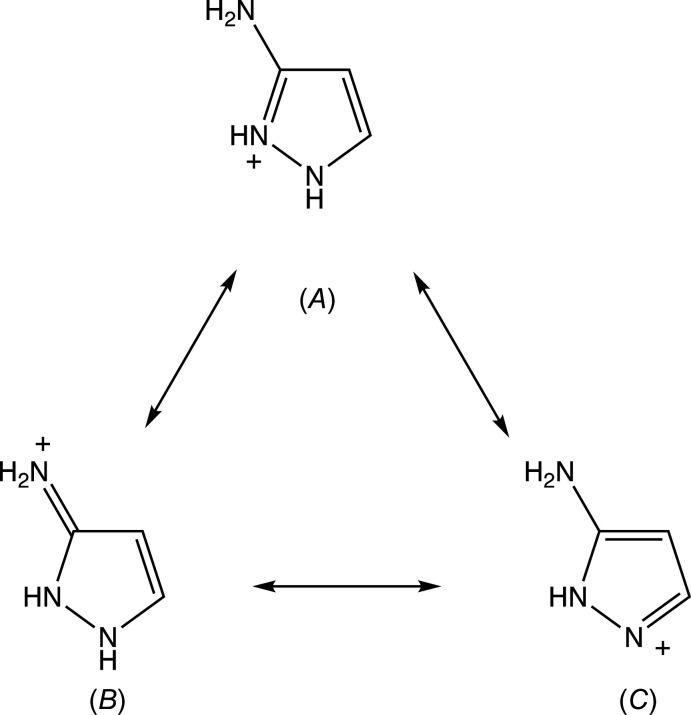
The three canonical forms that contribute to the electronic structure of the cations in compounds (I)–(III).

**Figure 5:**
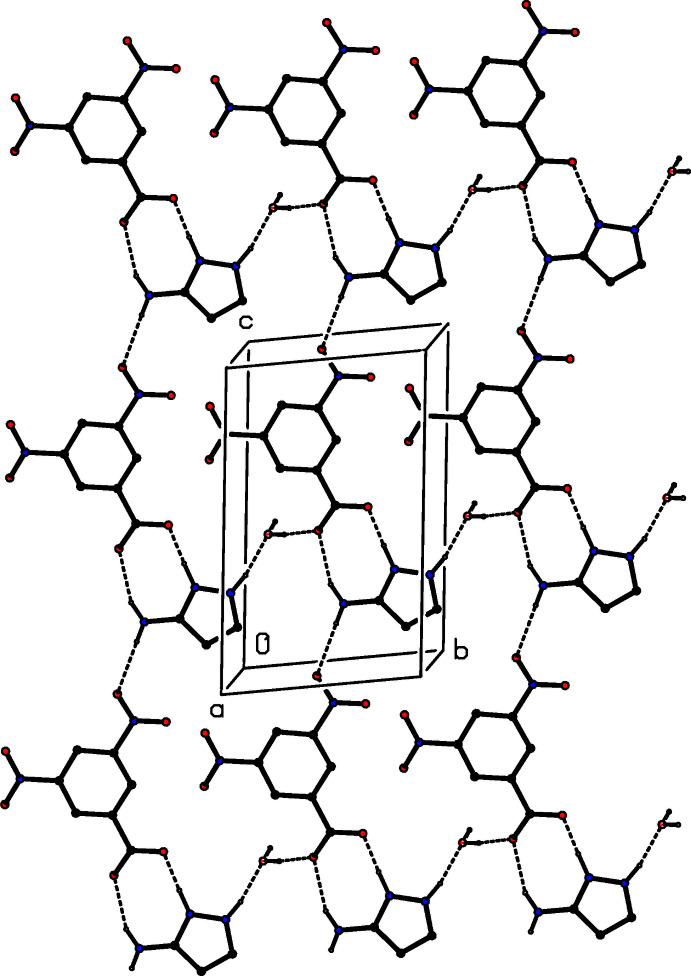
Part of the crystal structure of compound (I), showing the formation of a hydrogen-bonded sheet lying parallel to (100). Hydrogen bonds are drawn as dashed lines and, for the sake of clarity, the H atoms bonded to C atoms have been omitted.

**Figure 6:**
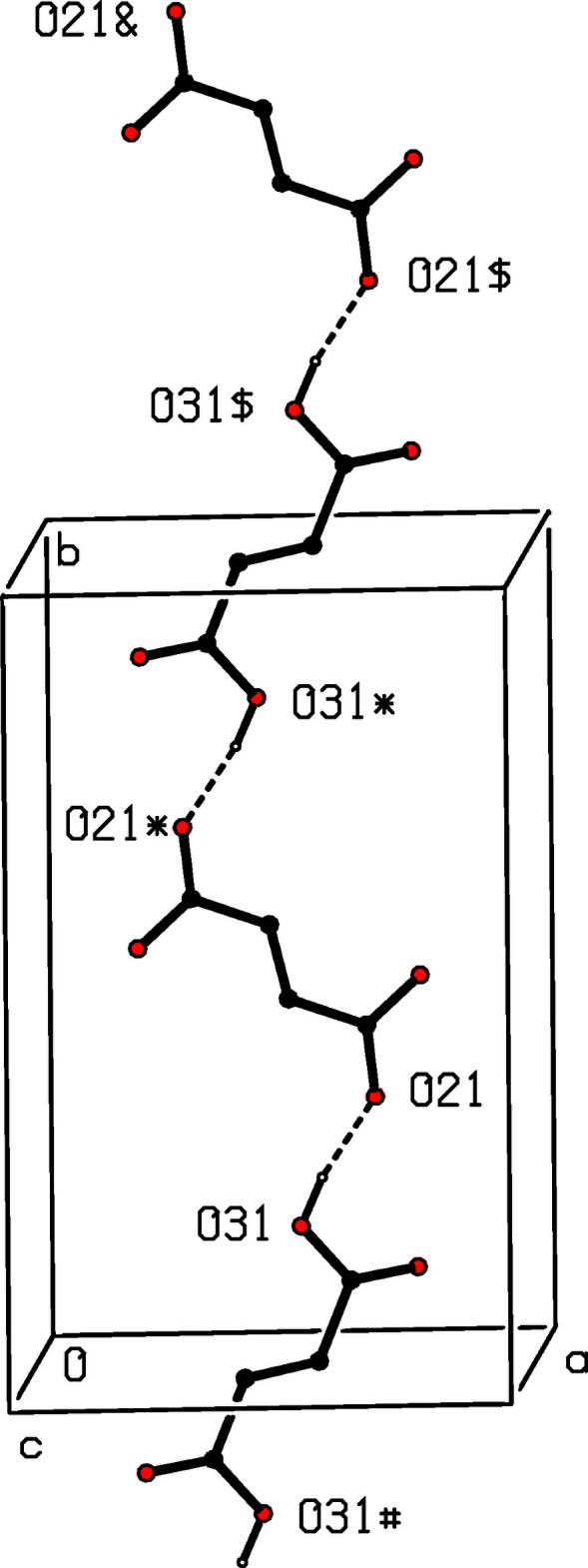
Part of the crystal structure of compound (II), showing the formation of a chain of alternating fumarate ions and fumaric acid mol­ecules. Hydrogen bonds are drawn as dashed lines and, for the sake of clarity, the cations and the H atoms bonded to C atoms have been omitted. The atoms marked with an asterisk (*), a hash (#), a dollar sign ($) or an ampersand (&) are at the symmetry positions (1 − *x*, 1 − *y*, 1 − *z*), (1 − *x*, −*y*, 1 − *z*), (*x*, 1 + *y*, *z*) and (1 − *x*, 2 − *y*, 1 − *z*), respectively. The atoms O21 and O31 (without symmetry symbols) are components of the reference species at (*x*, *y*, *z*).

**Figure 7:**
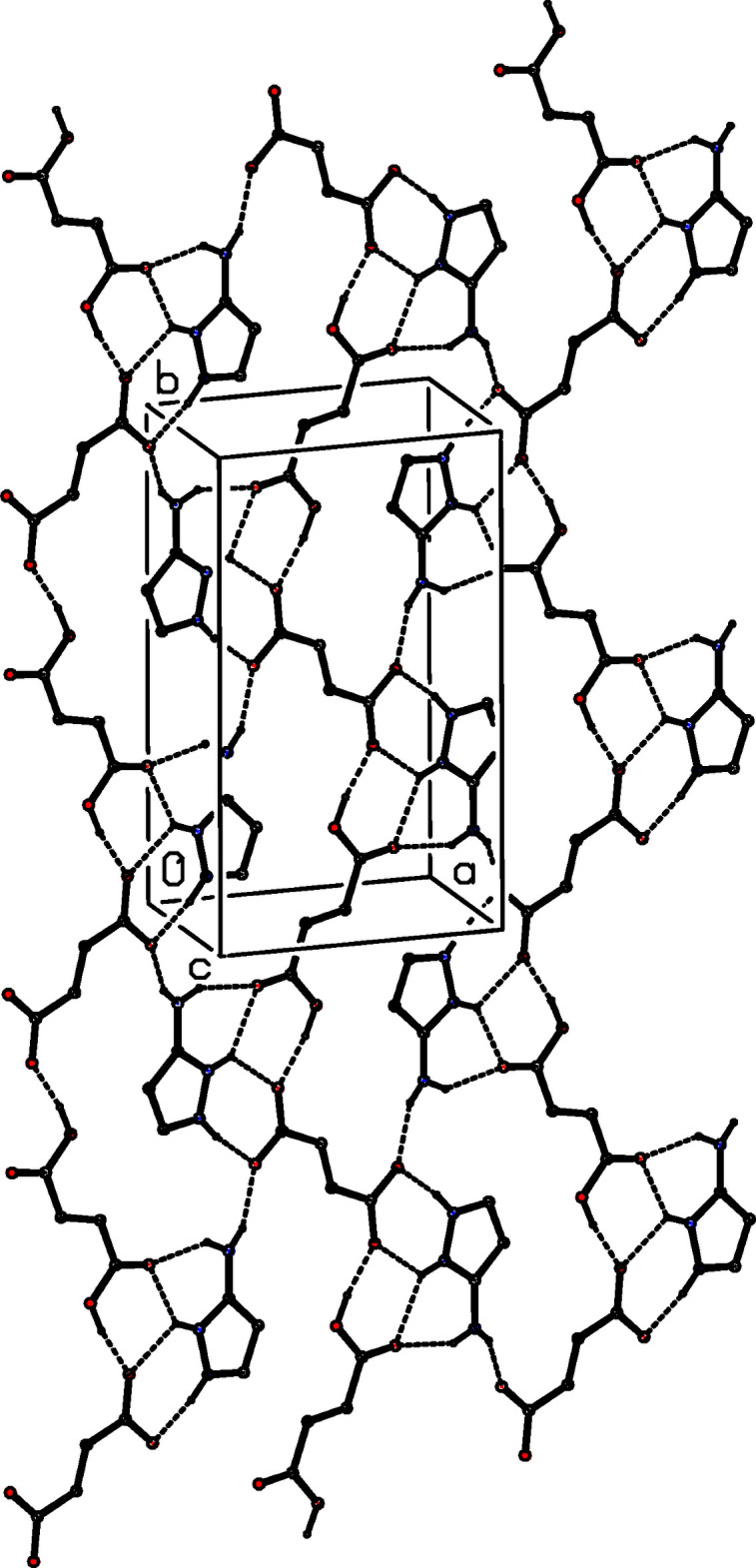
Part of the crystal structure of compound (II), showing the formation of a sheet lying parallel to (102). Hydrogen bonds are drawn as dashed lines and, for the sake of clarity, the cations and the H atoms bonded to C atoms have been omitted.

**Figure 8:**
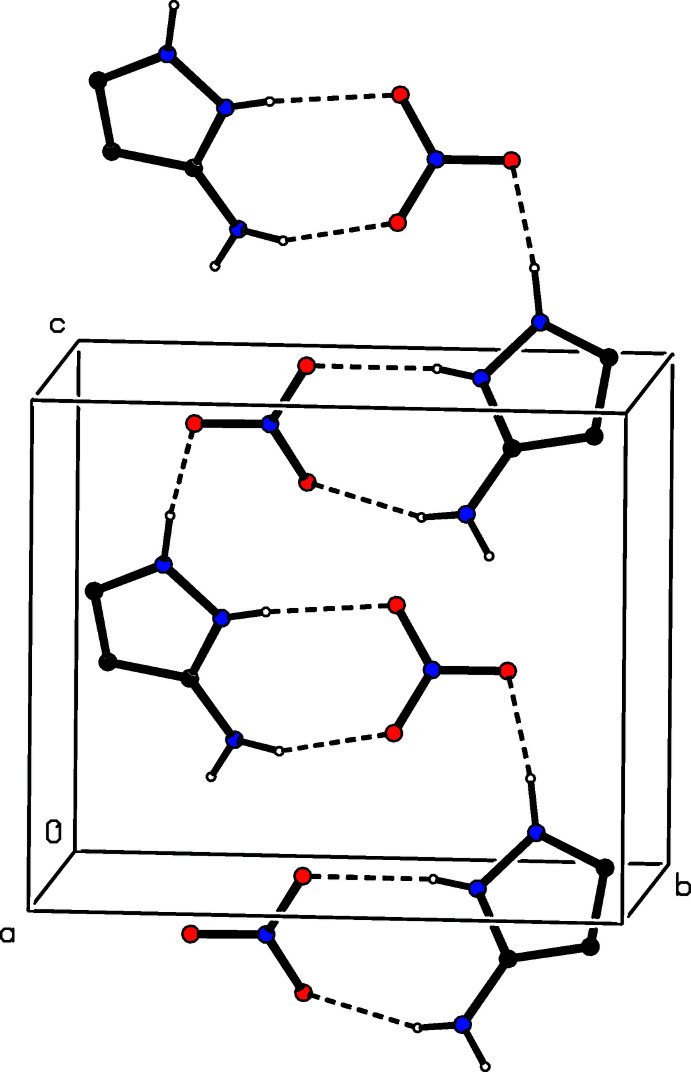
Part of the crystal structure of compound (III), showing the formation of a chain of rings running parallel to [001]. Hydrogen bonds are drawn as dashed lines and, for the sake of clarity, the H atoms bonded to C atoms have been omitted.

**Figure 9:**
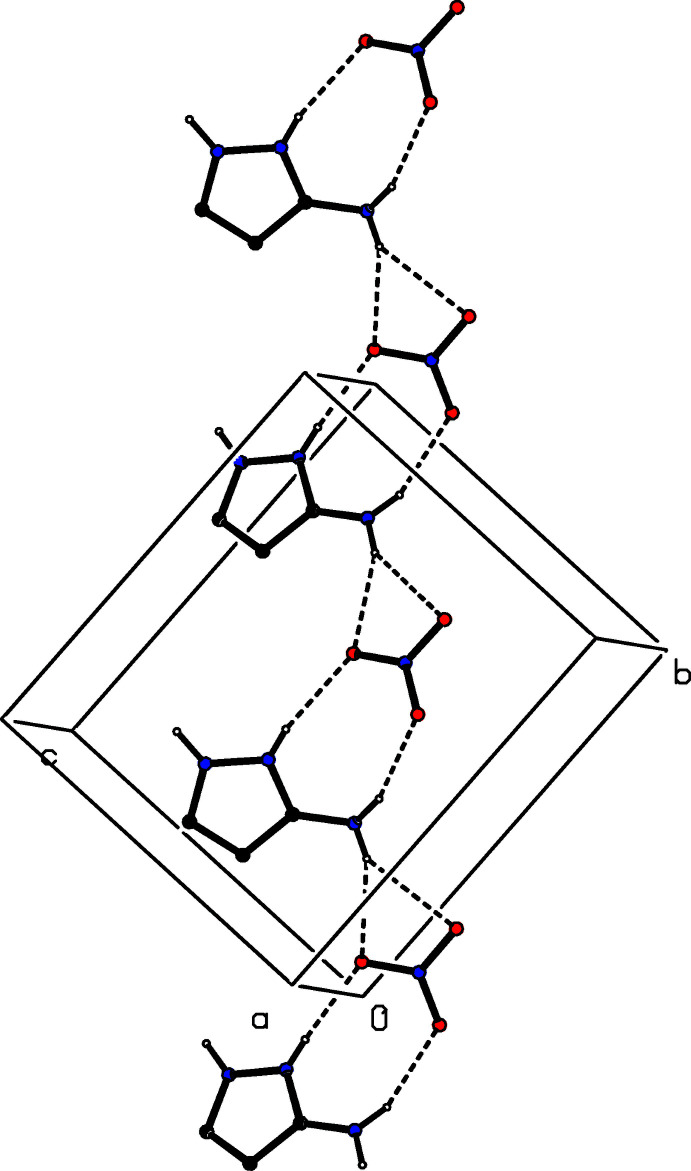
Part of the crystal structure of compound (III), showing the formation of a chain of rings running parallel to [011]. Hydrogen bonds are drawn as dashed lines and, for the sake of clarity, the H atoms bonded to C atoms have been omitted.

**Figure 10:**
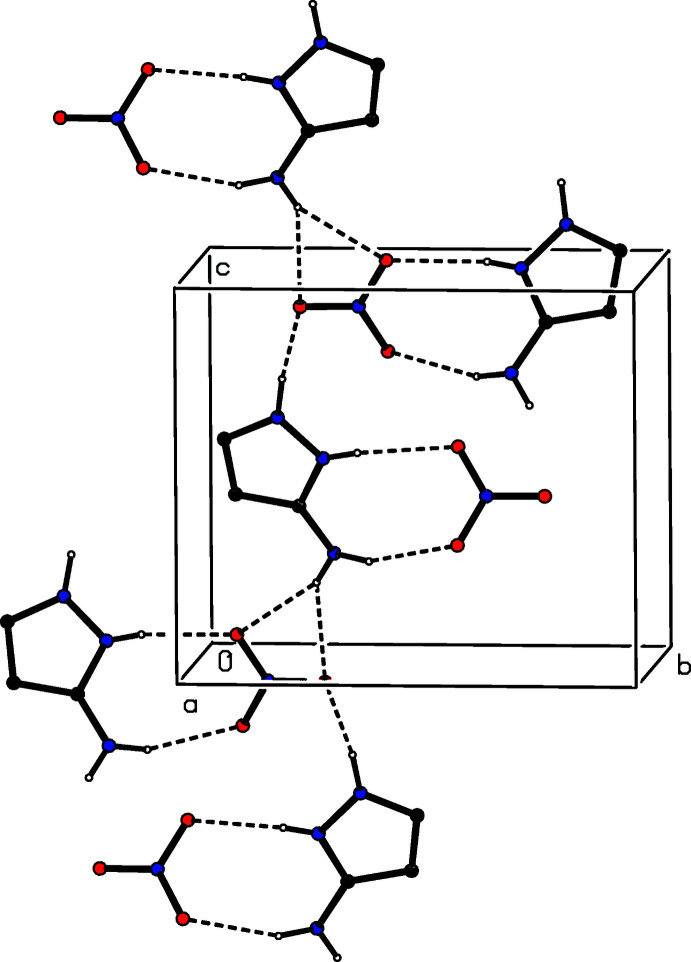
Part of the crystal structure of compound (III), showing the formation of a chain of rings running parallel to [102]. Hydrogen bonds are drawn as dashed lines and, for the sake of clarity, the H atoms bonded to C atoms have been omitted.

The ionic components in compound (III) are linked by two N—H⋯O hydrogen bonds to form an ion pair containing an 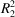
(8) ring (Fig. 3 ▸). Ion pairs of this type are linked by one two-centre N—H⋯O hydrogen bond and one three-centre N—H⋯(O)_2_ system into a three-dimensional framework structure, whose formation is readily analysed in terms of three simple one-dimensional sub-structures (Ferguson *et al.*, 1998*a* ▸,*b* ▸; Gregson *et al.*, 2000 ▸). The two-centre N—H⋯O hydrogen bond, acting alone, links ion pairs that are related by the 2_1_ screw axis along [001], forming a 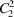
(7)
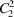
(9)[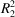
(8) chain of rings running parallel to [001] (Fig. 8 ▸). The three-centre N—H⋯(O)_2_ hydrogen bond links ion pairs that are related by the *n*-glide plane to form a chain of alternating 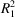
(4) and 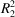
(8) rings running parallel to the [011] direction (Fig. 9 ▸). When the two-centre and three-centre systems act alternately, they link the ion pairs into a chain of rings running parallel to the [102] direction (Fig. 10 ▸). The combination of the chains along [001], [011] and [102] suffices to link all of the components into a three-dimensional framework structure.

## Database survey   

**Figure 11:**
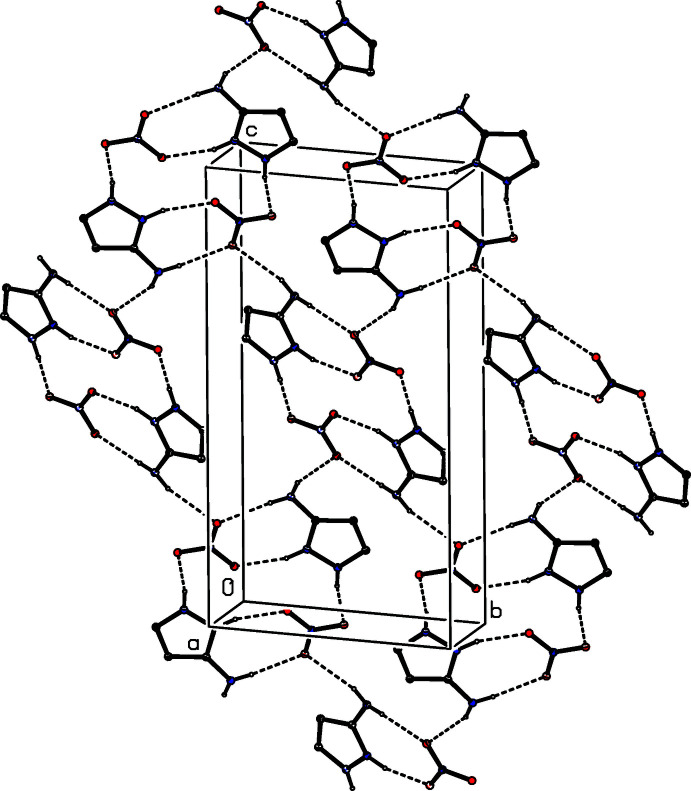
Part of the crystal structure of the monoclinic polymorph (III*a*) of 3-amino-1*H*-pyrazol-2-ium nitrate showing the formation of a hydrogen-bonded sheet parallel to (10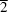). The deposited coordinates (Yamuna *et al.*, 2020 ▸) have been used. Hydrogen bonds are drawn as dashed lines and, for the sake of clarity, the H atoms bonded to C atoms have been omitted.

As noted above in Section 2, a monoclinic polymorph of the nitrate salt, denoted (III*a*) has recently been reported, but without any analysis or description of the supra­molecular assembly (Yamuna *et al.*, 2020 ▸). As found in the ortho­rhom­bic polymorph (III), the ions in (III*a*) are linked by two N—H⋯O hydrogen bonds to form an ion pair characterized by an 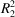
(8) motif. Two further N—H⋯O hydrogen bonds link these ion pairs into a sheet lying parallel to (10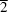), in which rings of 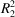
(8), 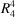
(14) and 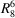
(26) types are present (Fig. 11 ▸). Sheets of this type are linked by a C—H⋯O hydrogen bond to form a three-dimensional framework structure. In the picrate salt, the ions are linked into sheets by a combination of N—H⋯O and C—H⋯O hydrogen bonds (Infantes *et al.*, 1999 ▸). In the hydrogen succinate salt, a combination of O—H⋯O and N—H⋯O hydrogen bonds links the ions into sheets containing 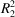
(8), 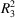
(12) and 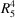
(20) rings (Yamuna *et al.*, 2014 ▸). The structure of the tri­fluoro­acetate, which crystallizes with *Z*′ = 2, and with disorder in each of the independent anions, contains only N—H⋯O hydrogen bonds, which link the ions into complex sheets (Yamuna *et al.*, 2013 ▸). We also note that the structure of tetra­kis­(3-amino-1*H*-pyrazol-2-ium) bis­(μ-chloro)­octa­chloro­dibismuth, (C_3_H_6_N_3_)_4_(Bi_2_Cl_10_), has been reported (Ferjani & Boughzala, 2018 ▸).

A number of structures have been reported in which fumarate dianions co-exist in equal numbers with neutral fumaric acid mol­ecules, as found here for compound (II). Recently reported examples include the salts formed with 2-amino-5-methyl­pyridine (Hemamalini & Fun, 2010 ▸), *N*,*N*′,*N*′′-triisoprop­ylguanidine (Said *et al.*, 2012 ▸), 2-amino­pyridine (Dong *et al.*, 2013 ▸; Solovyov, 2016 ▸) and di-*n*-butyl­amine (Tang *et al.*, 2015 ▸). We also note a rather earlier report on the structure of a salt formed by [tris­(phenan­thro­line)cobalt(II)] in which all three possible forms fumarate(2−), hydrogenfumarate(1−) and neutral fumaric acid are present in the molar ratio 1:2:3 (Liu *et al.*, 2003 ▸).

## Synthesis and crystallization   

The synthesis of compounds (I)–(III) employed commercially available 3-amino-1*H*-pyrazole, which was used as received. For the synthesis of compounds (I) and (II), a solution of 3-amino-1*H*-pyrazole (100 mg, 1.20 mmol) in ethanol (10 ml) was mixed with a solution of the appropriate acid, 3,5-di­nitro­benzoic acid (255 mg, 20 mmol) for (I) or fumaric acid (139 mg, 1.20 mmol) for (II), also in methanol (10 ml): for (III), a dilute solution of nitric acid in methanol (1:3, *v*/*v*, 10 ml) was added to a solution of 3-amino-1*H*-pyrazole (100 mg, 1.20 mmol) in ethanol (10 ml). Each of these mixtures was stirred at ambient temperature for 15 min and then set aside to crystallize at ambient temperature and in the presence of air. After one week, the resulting crystals were collected by filtration and dried in air: m.p. (I) 418–423 K, (II) 383–388 K, (III) 385–390 K. Crystals suitable for single-crystal X-ray diffraction were selected directly from the prepared samples.

## Refinement   

Crystal data, data collection and refinement details are summarized in Table 3 ▸. For compound (I), one low-angle reflection (1,0,0) that had been attenuated by the beam stop was omitted from the refinement. All H atoms were located in difference maps. The H atoms bonded to C atoms were then treated as riding atoms in geometrically idealized positions with C—H distances 0.93 Å and *U*_iso_(H) = 1.2*U*_eq_(C). For the H atoms bonded to N or O atoms, the atomic coordinates were refined with *U*_iso_(H) = 1.2*U*_eq_(N) or 1.5*U*_eq_(O). In the absence of significant resonant scattering, it was not possible to determine the correct orientation of the structure of (III) relative to the polar axis direction, although this has no chemical significance.

## Supplementary Material

Crystal structure: contains datablock(s) global, I, II, III. DOI: 10.1107/S2056989020015959/dx2033sup1.cif

Structure factors: contains datablock(s) I. DOI: 10.1107/S2056989020015959/dx2033Isup2.hkl

Structure factors: contains datablock(s) II. DOI: 10.1107/S2056989020015959/dx2033IIsup3.hkl

Structure factors: contains datablock(s) III. DOI: 10.1107/S2056989020015959/dx2033IIIsup4.hkl

Supporting information file. DOI: 10.1107/S2056989020015959/dx2033Isup5.cml

Supporting information file. DOI: 10.1107/S2056989020015959/dx2033IIIsup6.cml

CCDC references: 2048572, 2048571, 2048570

Additional supporting information:  crystallographic information; 3D view; checkCIF report

## Figures and Tables

**Table 1:** Selected bond distances (Å) The data for the monoclinic polymorph (III*a*) are taken from Yamuna *et al.* (2020 ▸), but with the atom labels adjusted to match those used for (I)–(III).

Parameter	(I)	(II)	(III)	(III*a*)
N11—N12	1.362 (2)	1.3467 (17)	1.351 (4)	1.358 (2)
N12—C13	1.338 (2)	1.3340 (17)	1.336 (4)	1.347 (2)
C13—C14	1.402 (2)	1.391 (2)	1.393 (4)	1.403 (3)
C14—C15	1.365 (3)	1.366 (2)	1.367 (5)	1.372 (2)
C15—N11	1.331 (2)	1.3187 (19)	1.334 (4)	1.329 (3)
C13—N131	1.348 (2)	1.3480 (19)	1.338 (4)	1.338 (2)

**Table 2:** Hydrogen bond parameters (Å, °)

Compound	*D*—H⋯*A*	*D*—H	H⋯*A*	*D*⋯*A*	*D*—H⋯*A*
(I)	O31—H31⋯O22^i^	0.88 (3)	1.87 (3)	2.746 (2)	175 (3)
	O31—H32⋯O21^ii^	0.75 (4)	2.36 (3)	2.989 (2)	143 (3)
	N131—H131⋯O22	0.88 (2)	2.08 (2)	2.920 (2)	159 (2)
	N131—H132⋯O25^iii^	0.82 (2)	2.31 (3)	3.128 (2)	171 (3)
	N11—H11⋯O31	0.89 (2)	1.83 (2)	2.707(2	169 (2)
	N12—H12⋯O21	1.00 (2)	1.60 (2)	2.5981 (19)	177.9 (18)
					
(II)	O31—H31⋯O21	0.90 (2)	1.65 (2)	2.5370 (15)	169 (2)
	N11—H11⋯O22	0.910 (18)	1.796 (17)	2.6989 (16)	171.4 (17)
	N12—H12⋯O21	0.892 (17)	2.172 (17)	2.8267 (17)	129.7(14
	N12—H12⋯O32	0.892 (17)	2.133 (17)	2.8641 (16)	138.6 (15)
	N131—H131⋯O32	0.82 (2)	2.33 (3)	3.052 (2)	148 (2)
	N131—H132⋯O22^iv^	0.908 (19)	2.03 (2)	2.8922 (18)	159.4 (17)
					
(III)	N11—H11⋯O23^v^	0.93 (5)	1.94 (5)	2.860 (4)	170 (3)
	N12—H12⋯O21	0.76 (4)	2.19 (4)	2.914 (3)	158 (3)
	N131—H131⋯O22	0.88 (5)	2.16 (5)	3.001 (4)	159 (4)
	N131—H132⋯O21^vi^	0.81 (5)	2.36 (4)	3.126 (4)	157 (4)
	N131—H132⋯O23^vi^	0.81 (5)	2.50 (5)	3.223 (4)	148 (4)

Symmetry codes: (i) *x*, 1 + *y*, *z*; (ii) −*x*, 2 − *y*, 1 − *z*; (iii) *x*, *y*, −1 + *z*; (iv) 2 − *x*, −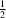 + *y*, 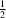 − *z*; (v) 1 − *x*, 1 − *y*, 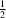 + *z*; (vi) 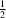 − *x*, −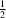 + *y*, −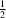 + *z*.

**Table 3:** Experimental details

	(I)	(II)	(III)
Crystal data			
Chemical formula	C_7_H_3_N_2_O_6_^+^·C_3_H_6_N_3_^−^·H_2_O	2C_3_H_6_N_3_^+^·C_4_H_2_O_4_^2−^·C_4_H_4_O_4_	C_3_H_6_N_3_^+^·NO_3_^−^
*M*_r_	313.24	398.34	146.12
Crystal system, space group	Triclinic, *P* 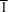	Monoclinic, *P*2_1_/*c*	Orthorhombic, *P* *n* *a*2_1_
Temperature (K)	296	296	296
*a*, *b*, *c* (Å)	6.6864 (7), 8.1857 (9), 12.649 (1)	8.5410 (4), 14.0507 (7), 7.5137 (4)	7.270 (1), 9.907 (2), 8.551 (2)
α, β, γ (°)	79.424 (9), 85.583 (9), 75.586 (9)	90, 98.827 (6), 90	90, 90, 90
*V* (Å^3^)	658.78 (12)	891.02 (8)	615.9 (2)
*Z*	2	2	4
Radiation type	Mo *K*α	Mo *K*α	Mo *K*α
μ (mm^−1^)	0.14	0.12	0.14
Crystal size (mm)	0.50 × 0.40 × 0.04	0.44 × 0.38 × 0.30	0.50 × 0.24 × 0.20

Data collection			
Diffractometer	Oxford Diffraction Xcalibur with Sapphire CCD	Oxford Diffraction Xcalibur with Sapphire CCD	Oxford Diffraction Xcalibur with Sapphire CCD
Absorption correction	Multi-scan (*CrysAlis RED*; Oxford Diffraction, 2009 ▸)	Multi-scan (*CrysAlis RED*; Oxford Diffraction, 2009 ▸)	Multi-scan (*CrysAlis RED*; Oxford Diffraction, 2009 ▸)
*T*_min_, *T*_max_	0.886, 0.995	0.897, 0.964	0.911, 0.973
No. of measured, independent and observed [*I* > 2σ(*I*)] reflections	4597, 2805, 2093	3658, 1909, 1413	2221, 942, 806
*R*_int_	0.014	0.016	0.020
(sin θ/λ)_max_ (Å^−1^)	0.651	0.651	0.656

Refinement			
*R*[*F* ^2^ > 2σ(*F* ^2^)], *wR*(*F* ^2^), *S*	0.044, 0.114, 1.04	0.037, 0.110, 1.07	0.036, 0.092, 1.11
No. of reflections	2805	1909	942
No. of parameters	217	143	103
No. of restraints	0	0	1
H-atom treatment	H atoms treated by a mixture of independent and constrained refinement	H atoms treated by a mixture of independent and constrained refinement	H atoms treated by a mixture of independent and constrained refinement
Δρ_max_, Δρ_min_ (e Å^−3^)	0.21, −0.23	0.16, −0.17	0.15, −0.15

Computer programs: *CrysAlis CCD* (Oxford Diffraction, 2009 ▸), *CrysAlis RED* (Oxford Diffraction, 2009 ▸), *SHELXT* (Sheldrick, 2015*a* ▸), *SHELXL2014* (Sheldrick, 2015*b* ▸) and *PLATON* (Spek, 2020 ▸).

## supplementary crystallographic information

### 3-Amino-1H-pyrazol-2-ium 3,5-dinitrobenzoate monohydrate (I) Crystal data

**Table:**

C_7_H_3_N_2_O_6_^+^·C_3_H_6_N_3_^−^·H_2_O	*Z* = 2
*M**_r_* = 313.24	*F*(000) = 324
Triclinic, *P*1	*D*_x_ = 1.579 Mg m^−^^3^
*a* = 6.6864 (7) Å	Mo *K*α radiation, λ = 0.71073 Å
*b* = 8.1857 (9) Å	Cell parameters from 2812 reflections
*c* = 12.649 (1) Å	θ = 2.6–27.8°
α = 79.424 (9)°	µ = 0.14 mm^−^^1^
β = 85.583 (9)°	*T* = 296 K
γ = 75.586 (9)°	Plate, yellow
*V* = 658.78 (12) Å^3^	0.50 × 0.40 × 0.04 mm

### 3-Amino-1H-pyrazol-2-ium 3,5-dinitrobenzoate monohydrate (I) Data collection

**Table:**

Oxford Diffraction Xcalibur with Sapphire CCD diffractometer	2805 independent reflections
Radiation source: Enhance (Mo) X-ray Source	2093 reflections with *I* > 2σ(*I*)
Graphite monochromator	*R*_int_ = 0.014
ω scans	θ_max_ = 27.5°, θ_min_ = 2.6°
Absorption correction: multi-scan (CrysAlis RED; Oxford Diffraction, 2009)	*h* = −8→8
*T*_min_ = 0.886, *T*_max_ = 0.995	*k* = −5→10
4597 measured reflections	*l* = −16→16

### 3-Amino-1H-pyrazol-2-ium 3,5-dinitrobenzoate monohydrate (I) Refinement

**Table:**

Refinement on *F*^2^	Primary atom site location: difference Fourier map
Least-squares matrix: full	Hydrogen site location: mixed
*R*[*F*^2^ > 2σ(*F*^2^)] = 0.044	H atoms treated by a mixture of independent and constrained refinement
*wR*(*F*^2^) = 0.114	*w* = 1/[σ^2^(*F*_o_^2^) + (0.0501*P*)^2^ + 0.2176*P*] where *P* = (*F*_o_^2^ + 2*F*_c_^2^)/3
*S* = 1.04	(Δ/σ)_max_ < 0.001
2805 reflections	Δρ_max_ = 0.21 e Å^−^^3^
217 parameters	Δρ_min_ = −0.23 e Å^−^^3^
0 restraints	

### 3-Amino-1H-pyrazol-2-ium 3,5-dinitrobenzoate monohydrate (I) Special details

**Table:**

Geometry. All esds (except the esd in the dihedral angle between two l.s. planes) are estimated using the full covariance matrix. The cell esds are taken into account individually in the estimation of esds in distances, angles and torsion angles; correlations between esds in cell parameters are only used when they are defined by crystal symmetry. An approximate (isotropic) treatment of cell esds is used for estimating esds involving l.s. planes.

### 3-Amino-1H-pyrazol-2-ium 3,5-dinitrobenzoate monohydrate (I) Fractional atomic coordinates and isotropic or equivalent isotropic displacement parameters (Å^2^)

**Table:**

	*x*	*y*	*z*	*U*_iso_*/*U*_eq_	
N11	0.1394 (3)	0.94340 (19)	0.24519 (12)	0.0395 (4)	
H11	0.113 (3)	1.014 (3)	0.2934 (17)	0.047*	
N12	0.1901 (2)	0.77011 (18)	0.27559 (11)	0.0341 (3)	
H12	0.208 (3)	0.716 (2)	0.3528 (16)	0.041*	
C13	0.2255 (3)	0.6994 (2)	0.18655 (13)	0.0337 (4)	
C14	0.1959 (3)	0.8317 (2)	0.09726 (14)	0.0427 (4)	
H14	0.2097	0.8204	0.0250	0.051*	
C15	0.1428 (3)	0.9803 (2)	0.13825 (15)	0.0449 (5)	
H15	0.1136	1.0900	0.0978	0.054*	
N131	0.2838 (3)	0.5283 (2)	0.19133 (15)	0.0499 (5)	
H131	0.293 (4)	0.462 (3)	0.255 (2)	0.060*	
H132	0.293 (4)	0.493 (3)	0.134 (2)	0.060*	
C21	0.2746 (3)	0.3820 (2)	0.60721 (13)	0.0309 (4)	
C22	0.3285 (3)	0.2041 (2)	0.62773 (14)	0.0344 (4)	
H22	0.3511	0.1424	0.5713	0.041*	
C23	0.3480 (3)	0.1200 (2)	0.73307 (14)	0.0346 (4)	
C24	0.3153 (3)	0.2053 (2)	0.81972 (14)	0.0347 (4)	
H24	0.3301	0.1471	0.8901	0.042*	
C25	0.2594 (3)	0.3818 (2)	0.79622 (13)	0.0335 (4)	
C26	0.2404 (3)	0.4721 (2)	0.69249 (13)	0.0335 (4)	
H26	0.2052	0.5911	0.6799	0.040*	
C27	0.2520 (3)	0.4756 (2)	0.49231 (13)	0.0335 (4)	
O21	0.2332 (2)	0.63592 (15)	0.47770 (10)	0.0440 (3)	
O22	0.2511 (2)	0.39164 (17)	0.42035 (10)	0.0500 (4)	
N23	0.4084 (3)	−0.0685 (2)	0.75456 (14)	0.0476 (4)	
O23	0.4093 (4)	−0.14396 (19)	0.67999 (14)	0.0821 (6)	
O24	0.4524 (3)	−0.13973 (19)	0.84632 (13)	0.0700 (5)	
N25	0.2209 (3)	0.4769 (2)	0.88647 (12)	0.0452 (4)	
O25	0.2652 (3)	0.3964 (2)	0.97622 (12)	0.0811 (6)	
O26	0.1477 (3)	0.63024 (19)	0.86825 (12)	0.0647 (5)	
O31	0.0837 (3)	1.1210 (2)	0.41012 (14)	0.0608 (5)	
H31	0.140 (5)	1.208 (4)	0.409 (2)	0.091*	
H32	−0.017 (5)	1.144 (4)	0.440 (3)	0.091*	

### 3-Amino-1H-pyrazol-2-ium 3,5-dinitrobenzoate monohydrate (I) Atomic displacement parameters (Å^2^)

**Table:**

	*U*^11^	*U*^22^	*U*^33^	*U*^12^	*U*^13^	*U*^23^
N11	0.0506 (10)	0.0299 (8)	0.0369 (8)	−0.0063 (7)	−0.0022 (7)	−0.0071 (6)
N12	0.0438 (9)	0.0300 (7)	0.0283 (7)	−0.0085 (6)	−0.0021 (6)	−0.0045 (6)
C13	0.0346 (9)	0.0390 (9)	0.0286 (8)	−0.0084 (7)	−0.0026 (7)	−0.0088 (7)
C14	0.0484 (12)	0.0495 (11)	0.0282 (9)	−0.0092 (9)	−0.0051 (8)	−0.0038 (8)
C15	0.0502 (12)	0.0409 (11)	0.0384 (10)	−0.0082 (9)	−0.0069 (8)	0.0049 (8)
N131	0.0781 (13)	0.0369 (9)	0.0348 (9)	−0.0079 (8)	−0.0033 (9)	−0.0135 (7)
C21	0.0326 (9)	0.0321 (9)	0.0287 (8)	−0.0090 (7)	−0.0019 (7)	−0.0050 (6)
C22	0.0385 (10)	0.0327 (9)	0.0332 (9)	−0.0078 (7)	−0.0004 (7)	−0.0100 (7)
C23	0.0346 (10)	0.0276 (8)	0.0395 (9)	−0.0056 (7)	−0.0012 (7)	−0.0027 (7)
C24	0.0352 (10)	0.0375 (9)	0.0292 (8)	−0.0082 (7)	−0.0026 (7)	0.0001 (7)
C25	0.0356 (10)	0.0375 (9)	0.0293 (8)	−0.0086 (7)	−0.0014 (7)	−0.0106 (7)
C26	0.0384 (10)	0.0272 (8)	0.0337 (9)	−0.0055 (7)	−0.0021 (7)	−0.0047 (7)
C27	0.0372 (10)	0.0350 (9)	0.0287 (8)	−0.0098 (7)	−0.0007 (7)	−0.0053 (7)
O21	0.0691 (9)	0.0320 (7)	0.0302 (6)	−0.0130 (6)	−0.0038 (6)	−0.0016 (5)
O22	0.0813 (11)	0.0418 (7)	0.0299 (7)	−0.0185 (7)	−0.0023 (6)	−0.0082 (6)
N23	0.0546 (11)	0.0311 (8)	0.0531 (10)	−0.0081 (7)	0.0028 (8)	−0.0018 (7)
O23	0.1407 (18)	0.0345 (8)	0.0655 (11)	−0.0093 (9)	0.0088 (11)	−0.0153 (8)
O24	0.0983 (14)	0.0407 (8)	0.0623 (10)	−0.0127 (8)	−0.0172 (9)	0.0144 (7)
N25	0.0543 (11)	0.0494 (10)	0.0346 (8)	−0.0127 (8)	0.0017 (7)	−0.0148 (7)
O25	0.1324 (17)	0.0746 (12)	0.0301 (8)	−0.0065 (11)	−0.0122 (9)	−0.0142 (7)
O26	0.0950 (13)	0.0461 (9)	0.0547 (9)	−0.0108 (8)	0.0045 (8)	−0.0243 (7)
O31	0.0825 (13)	0.0466 (9)	0.0590 (10)	−0.0185 (9)	−0.0006 (8)	−0.0201 (7)

### 3-Amino-1H-pyrazol-2-ium 3,5-dinitrobenzoate monohydrate (I) Geometric parameters (Å, º)

**Table:**

N11—C15	1.331 (2)	C22—H22	0.9300
N11—N12	1.362 (2)	C23—C24	1.380 (2)
N11—H11	0.89 (2)	C23—N23	1.473 (2)
N12—C13	1.338 (2)	C24—C25	1.380 (2)
N12—H12	0.999 (19)	C24—H24	0.9300
C13—N131	1.348 (2)	C25—C26	1.381 (2)
C13—C14	1.402 (2)	C25—N25	1.468 (2)
C14—C15	1.365 (3)	C26—H26	0.9300
C14—H14	0.9300	C27—O22	1.238 (2)
C15—H15	0.9300	C27—O21	1.267 (2)
N131—H131	0.88 (2)	N23—O23	1.217 (2)
N131—H132	0.82 (3)	N23—O24	1.222 (2)
C21—C26	1.389 (2)	N25—O26	1.214 (2)
C21—C22	1.390 (2)	N25—O25	1.221 (2)
C21—C27	1.513 (2)	O31—H31	0.89 (3)
C22—C23	1.383 (2)	O31—H32	0.74 (3)
			
C15—N11—N12	108.82 (15)	C21—C22—H22	120.4
C15—N11—H11	129.5 (13)	C24—C23—C22	122.72 (15)
N12—N11—H11	121.7 (13)	C24—C23—N23	118.18 (15)
C13—N12—N11	108.10 (14)	C22—C23—N23	119.10 (16)
C13—N12—H12	130.2 (11)	C23—C24—C25	116.41 (15)
N11—N12—H12	121.5 (11)	C23—C24—H24	121.8
N12—C13—N131	121.65 (16)	C25—C24—H24	121.8
N12—C13—C14	108.13 (15)	C24—C25—C26	123.17 (15)
N131—C13—C14	130.21 (17)	C24—C25—N25	117.91 (15)
C15—C14—C13	105.77 (16)	C26—C25—N25	118.91 (15)
C15—C14—H14	127.1	C25—C26—C21	118.86 (15)
C13—C14—H14	127.1	C25—C26—H26	120.6
N11—C15—C14	109.19 (16)	C21—C26—H26	120.6
N11—C15—H15	125.4	O22—C27—O21	125.03 (16)
C14—C15—H15	125.4	O22—C27—C21	118.32 (15)
C13—N131—H131	118.8 (15)	O21—C27—C21	116.64 (14)
C13—N131—H132	116.5 (17)	O23—N23—O24	123.95 (17)
H131—N131—H132	124 (2)	O23—N23—C23	117.99 (17)
C26—C21—C22	119.64 (15)	O24—N23—C23	118.06 (17)
C26—C21—C27	120.66 (15)	O26—N25—O25	123.56 (17)
C22—C21—C27	119.70 (15)	O26—N25—C25	118.69 (15)
C23—C22—C21	119.19 (16)	O25—N25—C25	117.74 (16)
C23—C22—H22	120.4	H31—O31—H32	104 (3)
			
C15—N11—N12—C13	0.1 (2)	N25—C25—C26—C21	−179.30 (15)
N11—N12—C13—N131	178.47 (17)	C22—C21—C26—C25	−0.5 (3)
N11—N12—C13—C14	−0.1 (2)	C27—C21—C26—C25	178.99 (15)
N12—C13—C14—C15	0.1 (2)	C26—C21—C27—O22	−168.58 (17)
N131—C13—C14—C15	−178.3 (2)	C22—C21—C27—O22	10.9 (3)
N12—N11—C15—C14	0.0 (2)	C26—C21—C27—O21	10.7 (2)
C13—C14—C15—N11	0.0 (2)	C22—C21—C27—O21	−169.82 (16)
C26—C21—C22—C23	−0.3 (3)	C24—C23—N23—O23	170.02 (19)
C27—C21—C22—C23	−179.78 (16)	C22—C23—N23—O23	−10.7 (3)
C21—C22—C23—C24	0.3 (3)	C24—C23—N23—O24	−9.2 (3)
C21—C22—C23—N23	−179.00 (16)	C22—C23—N23—O24	170.15 (18)
C22—C23—C24—C25	0.5 (3)	C24—C25—N25—O26	−171.16 (18)
N23—C23—C24—C25	179.83 (15)	C26—C25—N25—O26	9.5 (3)
C23—C24—C25—C26	−1.4 (3)	C24—C25—N25—O25	9.0 (3)
C23—C24—C25—N25	179.28 (16)	C26—C25—N25—O25	−170.37 (19)
C24—C25—C26—C21	1.4 (3)		

### 3-Amino-1H-pyrazol-2-ium 3,5-dinitrobenzoate monohydrate (I) Hydrogen-bond geometry (Å, º)

**Table:**

*D*—H···*A*	*D*—H	H···*A*	*D*···*A*	*D*—H···*A*
O31—H31···O22^i^	0.88 (3)	1.87 (3)	2.746 (2)	175 (3)
O31—H32···O21^ii^	0.75 (4)	2.36 (3)	2.989 (2)	143 (3)
N131—H131···O22	0.88 (2)	2.08 (2)	2.920 (2)	159 (2)
N131—H132···O25^iii^	0.82 (2)	2.31 (3)	3.128 (2)	171 (3)
N11—H11···O31	0.89 (2)	1.83 (2)	2.707 (2)	169 (2)
N12—H12···O21	1.00 (2)	1.60 (2)	2.5981 (19)	177.9 (18)
N12—H12···O22	1.00 (2)	2.586 (17)	3.244 (2)	123.3 (13)

Symmetry codes: (i) *x*, *y*+1, *z*; (ii) −*x*, −*y*+2, −*z*+1; (iii) *x*, *y*, *z*−1.

### Bis(3-amino-1H-pyrazol-2-ium) fumarate–fumaric acid (1/1)2C3H6N3+·C4H2O42-·C4H4O4, (II). The reaction of 3-amino-1H-pyrazole with a dilute solution of nitric acid in methanol yields an second, orthorhombic polymorph of 3-amino-1H-pyrazol-2-ium nitrate, C3H6N3+·NO3- (II) Crystal data

**Table:**

2C_3_H_6_N_3_^+^·C_4_H_2_O_4_^2^^−^·C_4_H_4_O_4_	*F*(000) = 416
*M**_r_* = 398.34	*D*_x_ = 1.485 Mg m^−^^3^
Monoclinic, *P*2_1_/*c*	Mo *K*α radiation, λ = 0.71073 Å
*a* = 8.5410 (4) Å	Cell parameters from 1910 reflections
*b* = 14.0507 (7) Å	θ = 3.1–27.9°
*c* = 7.5137 (4) Å	µ = 0.12 mm^−^^1^
β = 98.827 (6)°	*T* = 296 K
*V* = 891.02 (8) Å^3^	Block, yellow
*Z* = 2	0.44 × 0.38 × 0.30 mm

### Bis(3-amino-1H-pyrazol-2-ium) fumarate–fumaric acid (1/1)2C3H6N3+·C4H2O42-·C4H4O4, (II). The reaction of 3-amino-1H-pyrazole with a dilute solution of nitric acid in methanol yields an second, orthorhombic polymorph of 3-amino-1H-pyrazol-2-ium nitrate, C3H6N3+·NO3- (II) Data collection

**Table:**

Oxford Diffraction Xcalibur with Sapphire CCD diffractometer	1909 independent reflections
Radiation source: Enhance (Mo) X-ray Source	1413 reflections with *I* > 2σ(*I*)
Graphite monochromator	*R*_int_ = 0.016
ω scans	θ_max_ = 27.5°, θ_min_ = 3.1°
Absorption correction: multi-scan (CrysAlis RED; Oxford Diffraction, 2009)	*h* = −10→8
*T*_min_ = 0.897, *T*_max_ = 0.964	*k* = −18→7
3658 measured reflections	*l* = −9→9

### Bis(3-amino-1H-pyrazol-2-ium) fumarate–fumaric acid (1/1)2C3H6N3+·C4H2O42-·C4H4O4, (II). The reaction of 3-amino-1H-pyrazole with a dilute solution of nitric acid in methanol yields an second, orthorhombic polymorph of 3-amino-1H-pyrazol-2-ium nitrate, C3H6N3+·NO3- (II) Refinement

**Table:**

Refinement on *F*^2^	Hydrogen site location: mixed
Least-squares matrix: full	H atoms treated by a mixture of independent and constrained refinement
*R*[*F*^2^ > 2σ(*F*^2^)] = 0.037	*w* = 1/[σ^2^(*F*_o_^2^) + (0.0632*P*)^2^ + 0.0649*P*] where *P* = (*F*_o_^2^ + 2*F*_c_^2^)/3
*wR*(*F*^2^) = 0.110	(Δ/σ)_max_ < 0.001
*S* = 1.07	Δρ_max_ = 0.16 e Å^−^^3^
1909 reflections	Δρ_min_ = −0.17 e Å^−^^3^
143 parameters	Extinction correction: SHELXL, Fc^*^=kFc[1+0.001xFc^2^λ^3^/sin(2θ)]^-1/4^
0 restraints	Extinction coefficient: 0.011 (3)
Primary atom site location: difference Fourier map	

### Bis(3-amino-1H-pyrazol-2-ium) fumarate–fumaric acid (1/1)2C3H6N3+·C4H2O42-·C4H4O4, (II). The reaction of 3-amino-1H-pyrazole with a dilute solution of nitric acid in methanol yields an second, orthorhombic polymorph of 3-amino-1H-pyrazol-2-ium nitrate, C3H6N3+·NO3- (II) Special details

**Table:**

Geometry. All esds (except the esd in the dihedral angle between two l.s. planes) are estimated using the full covariance matrix. The cell esds are taken into account individually in the estimation of esds in distances, angles and torsion angles; correlations between esds in cell parameters are only used when they are defined by crystal symmetry. An approximate (isotropic) treatment of cell esds is used for estimating esds involving l.s. planes.

### Bis(3-amino-1H-pyrazol-2-ium) fumarate–fumaric acid (1/1)2C3H6N3+·C4H2O42-·C4H4O4, (II). The reaction of 3-amino-1H-pyrazole with a dilute solution of nitric acid in methanol yields an second, orthorhombic polymorph of 3-amino-1H-pyrazol-2-ium nitrate, C3H6N3+·NO3- (II) Fractional atomic coordinates and isotropic or equivalent isotropic displacement parameters (Å^2^)

**Table:**

	*x*	*y*	*z*	*U*_iso_*/*U*_eq_	
N11	0.99750 (15)	0.35914 (9)	0.31532 (18)	0.0470 (3)	
H11	0.929 (2)	0.4025 (13)	0.350 (2)	0.056*	
N12	0.96429 (14)	0.26559 (9)	0.32111 (17)	0.0424 (3)	
H12	0.874 (2)	0.2459 (12)	0.355 (2)	0.051*	
N131	1.08253 (19)	0.11913 (10)	0.2752 (2)	0.0621 (4)	
H131	1.000 (3)	0.0955 (15)	0.298 (3)	0.074*	
H132	1.147 (2)	0.0843 (14)	0.215 (3)	0.074*	
C13	1.08323 (16)	0.21506 (10)	0.27297 (19)	0.0409 (3)	
C14	1.19608 (17)	0.27973 (11)	0.2328 (2)	0.0488 (4)	
H14	1.2919	0.2659	0.1943	0.059*	
C15	1.13683 (18)	0.36775 (11)	0.2619 (2)	0.0515 (4)	
H15	1.1872	0.4251	0.2462	0.062*	
C21	0.66915 (15)	0.41618 (9)	0.45088 (19)	0.0388 (3)	
O21	0.68480 (13)	0.32808 (7)	0.44357 (18)	0.0628 (4)	
O22	0.77359 (11)	0.47336 (7)	0.41820 (16)	0.0524 (3)	
C22	0.51841 (15)	0.45462 (9)	0.50057 (18)	0.0384 (3)	
H22	0.4460	0.4114	0.5345	0.046*	
C31	0.63476 (16)	0.10443 (10)	0.4570 (2)	0.0432 (4)	
O31	0.53859 (13)	0.17395 (8)	0.48097 (19)	0.0656 (4)	
H31	0.580 (3)	0.2323 (17)	0.471 (3)	0.098*	
O32	0.76437 (13)	0.11540 (7)	0.41269 (17)	0.0590 (3)	
C32	0.57247 (16)	0.00849 (10)	0.4868 (2)	0.0429 (4)	
H32	0.6409	−0.0430	0.4868	0.051*	

### Bis(3-amino-1H-pyrazol-2-ium) fumarate–fumaric acid (1/1)2C3H6N3+·C4H2O42-·C4H4O4, (II). The reaction of 3-amino-1H-pyrazole with a dilute solution of nitric acid in methanol yields an second, orthorhombic polymorph of 3-amino-1H-pyrazol-2-ium nitrate, C3H6N3+·NO3- (II) Atomic displacement parameters (Å^2^)

**Table:**

	*U*^11^	*U*^22^	*U*^33^	*U*^12^	*U*^13^	*U*^23^
N11	0.0421 (7)	0.0359 (7)	0.0670 (8)	0.0031 (5)	0.0209 (6)	−0.0044 (6)
N12	0.0325 (6)	0.0388 (7)	0.0599 (8)	0.0000 (5)	0.0200 (5)	−0.0029 (5)
N131	0.0584 (9)	0.0416 (8)	0.0929 (12)	0.0036 (6)	0.0326 (8)	−0.0120 (7)
C13	0.0358 (7)	0.0425 (8)	0.0462 (8)	0.0050 (6)	0.0116 (6)	−0.0062 (6)
C14	0.0350 (7)	0.0561 (10)	0.0596 (9)	0.0010 (6)	0.0208 (7)	−0.0072 (7)
C15	0.0449 (8)	0.0460 (9)	0.0683 (10)	−0.0069 (6)	0.0233 (7)	−0.0027 (7)
C21	0.0351 (7)	0.0283 (7)	0.0561 (8)	0.0010 (5)	0.0175 (6)	0.0006 (6)
O21	0.0511 (6)	0.0257 (5)	0.1215 (10)	0.0008 (4)	0.0448 (7)	−0.0020 (5)
O22	0.0398 (5)	0.0306 (5)	0.0944 (8)	−0.0007 (4)	0.0350 (5)	0.0023 (5)
C22	0.0330 (6)	0.0310 (6)	0.0551 (8)	−0.0008 (5)	0.0190 (6)	0.0013 (6)
C31	0.0381 (7)	0.0369 (8)	0.0576 (9)	−0.0018 (6)	0.0174 (6)	0.0022 (6)
O31	0.0489 (7)	0.0344 (6)	0.1225 (11)	−0.0015 (5)	0.0418 (7)	0.0041 (6)
O32	0.0444 (6)	0.0429 (6)	0.0973 (9)	−0.0043 (5)	0.0352 (6)	0.0035 (6)
C32	0.0391 (7)	0.0344 (7)	0.0584 (9)	0.0009 (6)	0.0179 (6)	0.0036 (6)

### Bis(3-amino-1H-pyrazol-2-ium) fumarate–fumaric acid (1/1)2C3H6N3+·C4H2O42-·C4H4O4, (II). The reaction of 3-amino-1H-pyrazole with a dilute solution of nitric acid in methanol yields an second, orthorhombic polymorph of 3-amino-1H-pyrazol-2-ium nitrate, C3H6N3+·NO3- (II) Geometric parameters (Å, º)

**Table:**

N11—C15	1.3187 (19)	C21—O21	1.2472 (17)
N11—N12	1.3467 (17)	C21—O22	1.2525 (15)
N11—H11	0.910 (18)	C21—C22	1.4953 (17)
N12—C13	1.3340 (16)	C22—C22^i^	1.313 (3)
N12—H12	0.888 (17)	C22—H22	0.9300
N131—C13	1.3480 (19)	C31—O32	1.2136 (17)
N131—H131	0.82 (2)	C31—O31	1.3065 (18)
N131—H132	0.90 (2)	C31—C32	1.4789 (19)
C13—C14	1.391 (2)	O31—H31	0.90 (2)
C14—C15	1.366 (2)	C32—C32^ii^	1.305 (3)
C14—H14	0.9300	C32—H32	0.9300
C15—H15	0.9300		
			
C15—N11—N12	107.69 (12)	N11—C15—H15	125.1
C15—N11—H11	132.6 (11)	C14—C15—H15	125.1
N12—N11—H11	119.7 (11)	O21—C21—O22	122.93 (12)
C13—N12—N11	109.74 (11)	O21—C21—C22	118.15 (11)
C13—N12—H12	129.7 (11)	O22—C21—C22	118.92 (12)
N11—N12—H12	120.5 (11)	C22^i^—C22—C21	124.31 (15)
C13—N131—H131	114.4 (15)	C22^i^—C22—H22	117.8
C13—N131—H132	122.1 (13)	C21—C22—H22	117.8
H131—N131—H132	119 (2)	O32—C31—O31	124.20 (14)
N12—C13—N131	121.59 (13)	O32—C31—C32	121.45 (13)
N12—C13—C14	107.03 (13)	O31—C31—C32	114.34 (12)
N131—C13—C14	131.33 (13)	C31—O31—H31	113.7 (15)
C15—C14—C13	105.71 (12)	C32^ii^—C32—C31	124.14 (17)
C15—C14—H14	127.1	C32^ii^—C32—H32	117.9
C13—C14—H14	127.1	C31—C32—H32	117.9
N11—C15—C14	109.82 (14)		
			
C15—N11—N12—C13	0.58 (17)	C13—C14—C15—N11	−0.23 (19)
N11—N12—C13—N131	177.04 (15)	O21—C21—C22—C22^i^	−174.42 (18)
N11—N12—C13—C14	−0.71 (17)	O22—C21—C22—C22^i^	5.4 (3)
N12—C13—C14—C15	0.57 (17)	O32—C31—C32—C32^ii^	−171.39 (19)
N131—C13—C14—C15	−176.88 (17)	O31—C31—C32—C32^ii^	8.1 (3)
N12—N11—C15—C14	−0.20 (19)		

Symmetry codes: (i) −*x*+1, −*y*+1, −*z*+1; (ii) −*x*+1, −*y*, −*z*+1.

### Bis(3-amino-1H-pyrazol-2-ium) fumarate–fumaric acid (1/1)2C3H6N3+·C4H2O42-·C4H4O4, (II). The reaction of 3-amino-1H-pyrazole with a dilute solution of nitric acid in methanol yields an second, orthorhombic polymorph of 3-amino-1H-pyrazol-2-ium nitrate, C3H6N3+·NO3- (II) Hydrogen-bond geometry (Å, º)

**Table:**

*D*—H···*A*	*D*—H	H···*A*	*D*···*A*	*D*—H···*A*
O31—H31···O21	0.90 (2)	1.65 (2)	2.5370 (15)	169 (2)
N11—H11···O21	0.910 (18)	2.527 (17)	3.0070 (17)	113.4 (13)
N11—H11···O22	0.910 (18)	1.796 (17)	2.6989 (16)	171.4 (17)
N12—H12···O21	0.892 (17)	2.172 (17)	2.8267 (17)	129.7 (14)
N12—H12···O32	0.892 (17)	2.133 (17)	2.8641 (16)	138.6 (15)
N131—H131···O32	0.82 (2)	2.33 (3)	3.052 (2)	148 (2)
N131—H132···O22^iii^	0.908 (19)	2.03 (2)	2.8922 (18)	159.4 (17)

Symmetry code: (iii) −*x*+2, *y*−1/2, −*z*+1/2.

### 3-Amino-1H-pyrazol-2-ium nitrate (III) Crystal data

**Table:**

C_3_H_6_N_3_^+^·NO_3_^−^	*D*_x_ = 1.576 Mg m^−^^3^
*M**_r_* = 146.12	Mo *K*α radiation, λ = 0.71073 Å
Orthorhombic, *P**n**a*2_1_	Cell parameters from 942 reflections
*a* = 7.270 (1) Å	θ = 3.2–27.8°
*b* = 9.907 (2) Å	µ = 0.14 mm^−^^1^
*c* = 8.551 (2) Å	*T* = 296 K
*V* = 615.9 (2) Å^3^	Needle, yellow
*Z* = 4	0.50 × 0.24 × 0.20 mm
*F*(000) = 304	

### 3-Amino-1H-pyrazol-2-ium nitrate (III) Data collection

**Table:**

Oxford Diffraction Xcalibur with Sapphire CCD diffractometer	942 independent reflections
Radiation source: Enhance (Mo) X-ray Source	806 reflections with *I* > 2σ(*I*)
Graphite monochromator	*R*_int_ = 0.020
ω scans	θ_max_ = 27.8°, θ_min_ = 3.2°
Absorption correction: multi-scan (CrysAlis RED; Oxford Diffraction, 2009)	*h* = −5→9
*T*_min_ = 0.911, *T*_max_ = 0.973	*k* = −12→12
2221 measured reflections	*l* = −11→4

### 3-Amino-1H-pyrazol-2-ium nitrate (III) Refinement

**Table:**

Refinement on *F*^2^	Primary atom site location: difference Fourier map
Least-squares matrix: full	Hydrogen site location: mixed
*R*[*F*^2^ > 2σ(*F*^2^)] = 0.036	H atoms treated by a mixture of independent and constrained refinement
*wR*(*F*^2^) = 0.092	*w* = 1/[σ^2^(*F*_o_^2^) + (0.055*P*)^2^ + 0.0228*P*] where *P* = (*F*_o_^2^ + 2*F*_c_^2^)/3
*S* = 1.11	(Δ/σ)_max_ < 0.001
942 reflections	Δρ_max_ = 0.15 e Å^−^^3^
103 parameters	Δρ_min_ = −0.15 e Å^−^^3^
1 restraint	

### 3-Amino-1H-pyrazol-2-ium nitrate (III) Special details

**Table:**

Geometry. All esds (except the esd in the dihedral angle between two l.s. planes) are estimated using the full covariance matrix. The cell esds are taken into account individually in the estimation of esds in distances, angles and torsion angles; correlations between esds in cell parameters are only used when they are defined by crystal symmetry. An approximate (isotropic) treatment of cell esds is used for estimating esds involving l.s. planes.

### 3-Amino-1H-pyrazol-2-ium nitrate (III) Fractional atomic coordinates and isotropic or equivalent isotropic displacement parameters (Å^2^)

**Table:**

	*x*	*y*	*z*	*U*_iso_*/*U*_eq_	
N11	0.4618 (4)	0.1812 (2)	0.6198 (4)	0.0508 (6)	
H11	0.503 (5)	0.195 (3)	0.721 (6)	0.061*	
N12	0.4203 (3)	0.2766 (2)	0.5126 (3)	0.0439 (6)	
H12	0.431 (4)	0.352 (4)	0.528 (4)	0.053*	
C13	0.3503 (3)	0.2185 (3)	0.3849 (3)	0.0397 (6)	
C14	0.3453 (4)	0.0800 (2)	0.4129 (4)	0.0445 (7)	
H14	0.3025	0.0135	0.3454	0.053*	
C15	0.4162 (4)	0.0616 (3)	0.5595 (5)	0.0522 (8)	
H15	0.4303	−0.0212	0.6092	0.063*	
N131	0.2968 (4)	0.2904 (3)	0.2604 (4)	0.0577 (7)	
H131	0.349 (5)	0.370 (5)	0.246 (5)	0.069*	
H132	0.264 (5)	0.249 (4)	0.183 (6)	0.069*	
N21	0.3928 (3)	0.6297 (2)	0.4171 (3)	0.0451 (6)	
O21	0.3536 (3)	0.56587 (19)	0.5376 (3)	0.0641 (7)	
O22	0.4480 (3)	0.57038 (18)	0.2981 (3)	0.0560 (6)	
O23	0.3784 (4)	0.75575 (19)	0.4165 (3)	0.0699 (7)	

### 3-Amino-1H-pyrazol-2-ium nitrate (III) Atomic displacement parameters (Å^2^)

**Table:**

	*U*^11^	*U*^22^	*U*^33^	*U*^12^	*U*^13^	*U*^23^
N11	0.0619 (14)	0.0457 (13)	0.0448 (15)	−0.0068 (10)	−0.0035 (13)	−0.0023 (12)
N12	0.0500 (13)	0.0315 (10)	0.0501 (15)	−0.0048 (9)	0.0029 (13)	−0.0092 (12)
C13	0.0375 (11)	0.0358 (12)	0.0457 (18)	−0.0010 (10)	0.0045 (11)	−0.0072 (13)
C14	0.0480 (13)	0.0321 (13)	0.0534 (18)	−0.0053 (11)	0.0010 (15)	−0.0091 (12)
C15	0.0575 (16)	0.0362 (13)	0.063 (2)	−0.0052 (12)	0.0044 (16)	0.0008 (14)
N131	0.0694 (16)	0.0474 (15)	0.0562 (18)	−0.0059 (12)	−0.0124 (14)	−0.0010 (13)
N21	0.0601 (13)	0.0324 (11)	0.0427 (13)	0.0020 (9)	−0.0002 (12)	0.0005 (12)
O21	0.1067 (18)	0.0398 (10)	0.0457 (13)	0.0027 (10)	0.0150 (14)	0.0043 (10)
O22	0.0818 (14)	0.0380 (10)	0.0482 (13)	−0.0002 (9)	0.0104 (12)	−0.0067 (10)
O23	0.1188 (18)	0.0274 (9)	0.0635 (16)	0.0089 (10)	0.0148 (15)	−0.0002 (11)

### 3-Amino-1H-pyrazol-2-ium nitrate (III) Geometric parameters (Å, º)

**Table:**

N11—C15	1.334 (4)	C14—H14	0.9300
N11—N12	1.351 (4)	C15—H15	0.9300
N11—H11	0.93 (5)	N131—H131	0.88 (4)
N12—C13	1.336 (4)	N131—H132	0.82 (5)
N12—H12	0.76 (4)	N21—O22	1.241 (4)
C13—N131	1.338 (4)	N21—O21	1.242 (3)
C13—C14	1.393 (4)	N21—O23	1.253 (3)
C14—C15	1.367 (5)		
			
C15—N11—N12	107.7 (3)	C13—C14—H14	126.9
C15—N11—H11	125 (2)	N11—C15—C14	109.3 (3)
N12—N11—H11	127 (2)	N11—C15—H15	125.4
C13—N12—N11	109.8 (2)	C14—C15—H15	125.4
C13—N12—H12	127 (3)	C13—N131—H131	118 (3)
N11—N12—H12	123 (3)	C13—N131—H132	117 (3)
N12—C13—N131	122.1 (2)	H131—N131—H132	118 (4)
N12—C13—C14	107.1 (3)	O22—N21—O21	120.9 (2)
N131—C13—C14	130.8 (3)	O22—N21—O23	119.7 (3)
C15—C14—C13	106.2 (3)	O21—N21—O23	119.4 (3)
C15—C14—H14	126.9		
			
C15—N11—N12—C13	−0.5 (3)	N131—C13—C14—C15	−179.6 (3)
N11—N12—C13—N131	179.8 (3)	N12—N11—C15—C14	0.1 (3)
N11—N12—C13—C14	0.6 (3)	C13—C14—C15—N11	0.3 (3)
N12—C13—C14—C15	−0.6 (3)		

### 3-Amino-1H-pyrazol-2-ium nitrate (III) Hydrogen-bond geometry (Å, º)

**Table:**

*D*—H···*A*	*D*—H	H···*A*	*D*···*A*	*D*—H···*A*
N11—H11···O22^i^	0.93 (5)	2.44 (3)	2.968 (3)	116 (3)
N11—H11···O23^i^	0.93 (5)	1.94 (5)	2.860 (4)	170 (3)
N12—H12···O21	0.76 (4)	2.19 (4)	2.914 (3)	158 (3)
N12—H12···O22^i^	0.76 (4)	2.59 (3)	3.029 (3)	119 (3)
N131—H131···O22	0.88 (5)	2.16 (5)	3.001 (4)	159 (4)
N131—H132···O21^ii^	0.81 (5)	2.36 (4)	3.126 (4)	157 (4)
N131—H132···O23^ii^	0.81 (5)	2.50 (5)	3.223 (4)	148 (4)

Symmetry codes: (i) −*x*+1, −*y*+1, *z*+1/2; (ii) −*x*+1/2, *y*−1/2, *z*−1/2.
